# Serum Neurofilament and Free Light Chain Levels in Patients Undergoing Treatment for Chronic Inflammatory Demyelinating Polyneuropathy

**DOI:** 10.3390/ijms25021254

**Published:** 2024-01-19

**Authors:** Marco Luigetti, Guido Primiano, Valerio Basile, Francesca Vitali, Stefano Pignalosa, Angela Romano, Andrea Sabino, Mariapaola Marino, Riccardo Di Santo, Gabriele Ciasca, Umberto Basile

**Affiliations:** 1 Dipartimento di Neuroscienze, Organi di Senso e Torace, Fondazione Policlinico Universitario Agostino Gemelli IRCCS, 00168 Rome, Italy; mluigetti@gmail.com (M.L.); angela.romano12@gmail.com (A.R.); 2 Dipartimento di Neuroscienze, Università Cattolica del Sacro Cuore, 00168 Rome, Italy; vitali.francesca95@gmail.com (F.V.); andrea.sabino@unicatt.it (A.S.); 3 Fondazione UILDM Lazio Onlus, 00167 Rome, Italy; 4 Clinical Pathology Unit and Cancer Biobank, Department of Research and Advanced Technologies, I.R.C.C.S. Regina Elena National Cancer Institute, 00144 Rome, Italy; valeriobasile90@gmail.com; 5 Dipartimento di Patologia Clinica, Ospedale Santa Maria Goretti, A.U.S.L. Latina, 04100 Latina, Italy; s.pignalosa@ausl.latina.it (S.P.); u.basile@ausl.latina.it (U.B.); 6 Sezione di Patologia Generale, Dipartimento di Medicina e Chirurgia Traslazionale, Università Cattolica del Sacro Cuore, 00168 Rome, Italy; mariapaola.marino@unicatt.it; 7 Fondazione Policlinico Universitario “A. Gemelli” I.R.C.C.S., 00168 Rome, Italy; riccardo.disanto92@gmail.com (R.D.S.); gabriele.ciasca@unicatt.it (G.C.); 8 Sezione di Fisica, Dipartimento di Neuroscienze, Università Cattolica del Sacro Cuore, 00168 Rome, Italy

**Keywords:** chronic inflammatory demyelinating polyneuropathy, biomarkers, neurofilament light chain, free light chains

## Abstract

Chronic inflammatory demyelinating polyradiculoneuropathy (CIDP) is an immune-mediated disorder affecting the peripheral nervous system. Despite the established diagnostic criteria, monitoring disease activity and treatment remains challenging. To address this limitation, we investigated serum neurofilament light chain (sNfL) and serum free light chains (sFLCs) as potential biomarkers. A total of 32 CIDP patients undergoing immunoglobulin therapy and 32 healthy controls enrolled in the present study, and agreed to have their blood plasma sNfL and sFLCs analyzed, while CIDP severity was assessed through the modified Rankin Scale (mRS) and the Overall Neuropathy Limitations Scale (ONLS). In line with the immunoglobulin treatment aimed at limiting neuronal damage administered to the majority of patients, sNfL levels did not exhibit significant differences between the two groups. However, CIDP patients showed significantly elevated sFLC and sFLC ratios, while the marker levels did not correlate with the clinical scores. The study confirms the potential of sFLCs as a sensitive biomarker of inflammatory processes in CIDP. Additionally, the present study results regarding neurofilaments strengthen the role of sNfL in monitoring CIDP treatments, confirming the effectiveness of immunoglobulin therapy. Overall, our results demonstrate how combining these markers can lead to better patient characterization for improved treatment.

## 1. Introduction

Chronic inflammatory demyelinating polyradiculoneuropathy (CIDP) is an acquired, immune-mediated inflammatory disorder characterized by demyelination of the peripheral nervous system (PNS). This condition results in progressive or recurrent, symmetrical, proximal, and distal weakness and sensory dysfunction in the extremities over a period of at least 2 months. The exact mechanisms underlying the pathogenesis of CIDP still remain not fully clear, but they presumably involve heterogeneous immune processes mediated by autoreactive T lymphocytes, B lymphocytes, complement components, inflammatory chemokines, and cytokines and antibodies to various glycoprotein and glycolipid nerve structures [1].

According to the 2021 EAN/PNS guidelines [2], the diagnosis of CIDP follows specific clinical and electrophysiological criteria, which may be supported by treatment response, neuroimaging findings, cerebrospinal fluid (CSF) analysis, or nerve biopsy. However, there is no evidence that these same tests are effective in monitoring disease activity and treatment response, mainly because of their limited ability to respond to the effects of disease-modifying interventions, especially in patients with severe nerve damage [3]. Currently, the evaluation of these parameters relies primarily on clinical outcome measurements, which often lead to premature treatment withdrawal or unnecessary treatment re-initiations. The need to address these shortcomings has led to a growing interest in the investigation of potential prognostic circulating CIPD biomarkers in recent years, with a particular focus on neurofilament light chain (NfL) [3,4,5,6].

Neurofilaments are major structural proteins of the neuronal cytoskeleton and are composed of three subunits: heavy (approximately 200 kDa), medium (approximately 160 kDa), and light (approximately 70 kDa) chains. These subunits contribute to the formation of the filamentous structure, providing structural support and maintaining the axonal caliber, which is crucial for proper neuronal function [7]. NfL is released into the extracellular space following axonal damage caused by various factors such as inflammation, neurodegeneration, trauma, and vascular injury. This release enables the detection of NfL in both cerebrospinal fluid (CSF) and blood, rendering this protein a valuable biomarker for assessing axonal damage in various central and peripheral nervous system disorders. The quantitative measurement of NfL levels has demonstrated considerable utility in the diagnosis, prognosis, and monitoring of disease progression in conditions such as multiple sclerosis, Alzheimer’s disease, and amyotrophic lateral sclerosis, among others [8].

Since an intense and protracted demyelination may result in axonal degeneration, especially of large myelinated nerve fibers [9], NfL might represent an indirect marker of disease progression and disease severity in CIDP. Consistently, Kapoor et al. found that intravenous immunoglobulin (IVIg) treatment effectively lowered serum neurofilament light chain (sNfL) levels in CIDP patients, bringing them closer to levels seen in healthy individuals [6].

Aside from NfL, other circulating and cerebrospinal fluid markers are starting to attract attention for a more in-depth characterization of neuroinflammatory diseases. For instance, in multiple sclerosis (MS), altered levels of free light chains (FLCs) have also been identified in both blood and CSF [10,11]. Notably, a recent meta-analysis by Arneth et al. has suggested the potential benefits of utilizing FLCs for the diagnosis of MS and clinically isolated syndrome (CIS) due to the efficiency and cost-effectiveness of their isolation and analysis [10]. Similarly, a consensus statement indicates that FLCs accumulate in the CSF in cases of chronic inflammatory diseases of the central nervous system [11]. These findings suggest that FLC levels in both blood and CSF may serve as general markers of neuroinflammation, a concept that could potentially extend to CIDP, although there is currently no scientific evidence to support this hypothesis. In this context, it is useful to provide more detailed information regarding the biogenesis and structure of these filaments. During the process of immunoglobulin production by plasma cells and lymphocytes, FLCs are secreted in higher quantities compared to heavy chains. The surplus light chains circulate in the bloodstream and are subsequently filtered by the glomerulus, eventually becoming detectable in urine [12].

The biological function of FLCs in chronic inflammatory diseases may involve the activation of the adaptive immune system, thereby triggering inflammation [13]. Elevated FLC levels have been observed in conditions strongly associated with inflammation, such as viral infections, systemic rheumatic diseases, and related disorders, often attributed to disturbances in the synthesis of hyperactive immunoglobulins as a consequence of immune system inflammatory responses [13].

FLCs have been found to be increased in autoimmune diseases as mini-autoantibodies of specific subsets of lymphocytes acting like main pathogenetic actors to disease activity [14,15]. The rapid turnover of FLCs represents a possible useful real-time surrogate marker of inflammation for assessing residual disease activity and B cell hyperactivity, in contrast to NfL. Kappa FLC (k_FLC_) gene rearrangement precedes the rearrangement of lambda FLC (λ_FLC_). Unlike the λ_FLC_ genes, this process for the k_FLC_ genes takes place normally in almost all peripheral B cells [16]. Regarding the physiological impact of elevated FLC levels, it has been suggested that FLCs are able to inhibit innate immune functions that can contribute to an increase in infections. k_FLC_ and λ_FLC_ showed a slight excess by the synthesis of immunoglobulin light chains, giving rise to a release of about 500 mg/day of FLCs in the peripheral blood [16]. FLCs can also be found in various body fluids, such as synovial fluid, CSF, urine, and saliva [16]. The production of an excess of protein without a reason or a specific function in a biological system is rare; FLCs should therefore be considered bioactive molecules rather than a secondary product of the synthesis of immunoglobulins [16]. The primary objective of this study was to comprehensively evaluate alterations in serum levels of NfL and FLCs in CIDP patients undergoing treatment to limit neuronal damage. The investigation specifically focused on establishing the correlation of these biomarkers with clinical outcomes, aiming to ascertain their efficacy as sensitive and easily accessible indicators for monitoring a patient’s treatment response and overall inflammatory status associated with this neurological disorder.

## 2. Results

The neurophysiological study, performed at the time of sample collection, confirmed the presence of axonal damage in all 32 CIDP patients. Serum immunofixation was negative in all patients, excluding paraproteinemic neuropathy. All patients underwent treatment for the management of CIDP to limit neuronal damage. Of the 32 patients enrolled, 25 were receiving IVIg therapy, and of these, 2 patients were also undergoing therapy with corticosteroids, and 1 patient was receiving both corticosteroids and immunosuppressive medication. Only one patient was receiving subcutaneous immunoglobulin (SCIg) therapy, and two patients were solely on corticosteroid therapy. Twenty-eight patients were in current treatment for a chronic progressive CIDP, while the remaining four were without therapy for a remission phase of the disease.

In Table 1, we summarize the demographic variables (sex and age), clinical variables (modified Rankin Scale (mRS) and Overall Neuropathy Limitations Scale (ONLS)), and the laboratory parameters (sNfL, k_FLC_, λ_FLC_, and their k_FLC_/λ_FLC_ ratio) measured in the 32 CIDP patients and 32 healthy controls. No statistically significant differences were observed between the median ages of the two groups (median difference of 3 years, *p* = 0.4) and in the sex distribution (*p* > 0.9).

**Figure 1:**
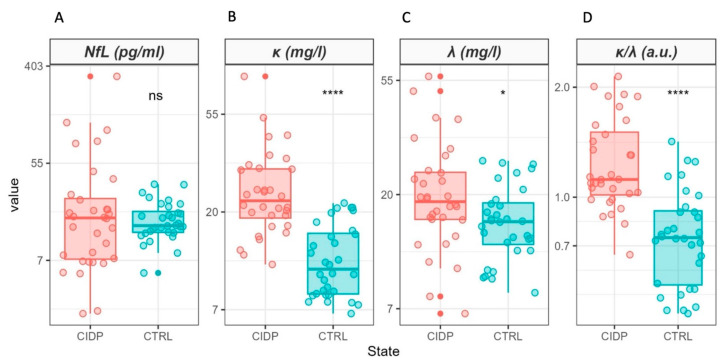
Box-plot analysis of NfL (**A**), kFLC (**B**), λFLC (**C**), and k/λ ratio (**D**) measured in CIDP patients (light red) and controls (light cyan). Comparisons between groups were performed using the Wilcoxon Unpaired Two-Sample Test. Statistical significance is indicated by the following symbols: ns *p* > 0.05; * *p* < 0.05; **** *p* < 0.0001. A logarithmic scale is used to enhance data visualization.

A box plot analysis of sNfL and FLC levels measured in the enrolled CIDP patients and controls is presented in Figure 1. The result of a Wilcoxon Mann–Whitney Test is superimposed on each plot. For clear visualization, sNfL expression levels are shown on a logarithmic scale, allowing the display of particularly high values, especially among the patients (Figure 1A). In this case, no significant statistical differences were observed between the two groups (*p* = 0.87), with similar median values but greater data dispersion in CIDP patients, likely associated with the competitive effects of the disease presence and the outcome of treatments.

**Figure 2:**
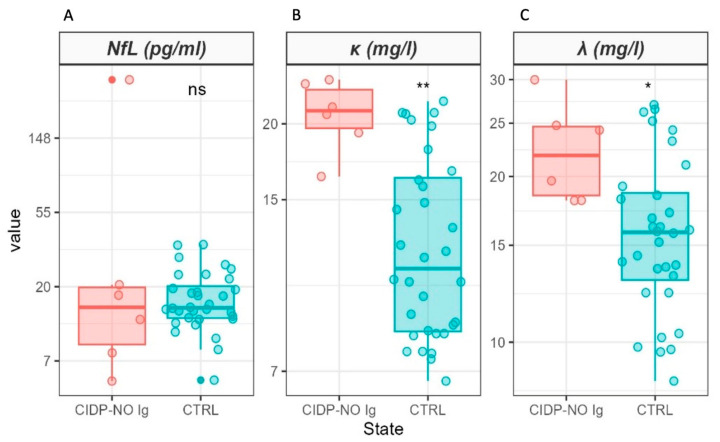
Box-plot analysis of NfL (**A**), kFLC (**B**), and λFLC (**C**) measured in CIDP patients not undergoing IVIg and SCIg (light red) and controls (light cyan). Comparisons between groups were performed using the Wilcoxon Unpaired Two-Sample Test. Statistical significance is indicated by the following symbols: ns *p* > 0.05; * *p* < 0.05; ** *p* < 0.01. A logarithmic scale is used to enhance data visualization.

Conversely, statistically significant increases in serum FLC levels were detected in CIDP patients compared to healthy controls, specifically k_FLC_ (Figure 1B, *p* = 7.0 × 10^−8^) and λ_FLC_ (Figure 1C, *p* = 0.022). Moreover, a higher value of the k_FLC_/λ_FLC_ ratio was observed in patients (Figure 1D, *p* = 5.7 × 10^−7^). To exclude the potential role of administered immunoglobulin in the increased FLC levels in the CIDP group, we compared serum FLC values in patients not treated with IVIg and SCIg at the time of sampling with those of healthy controls (Figure 2), and both k_FLC_ (Figure 2B, *p* = 0.002) and λ_FLC_ (Figure 2C, *p* = 0.017) were found to be significantly higher in the patient group.

Notably, compared to the standard reference levels, the vast majority of CIDP patients displayed k_FLC_ levels exceeding the reference range (3.3–19.4 mg/L), with only a few controls slightly exceeding this interval (Figure 1B). Despite similar significance levels (*p* = 5.5 × 10^−7^), only six patients and no controls exceeded the corresponding reference range for the k_FLC_/λ_FLC_ ratio (<0.26 or >1.65).

**Figure 3:**
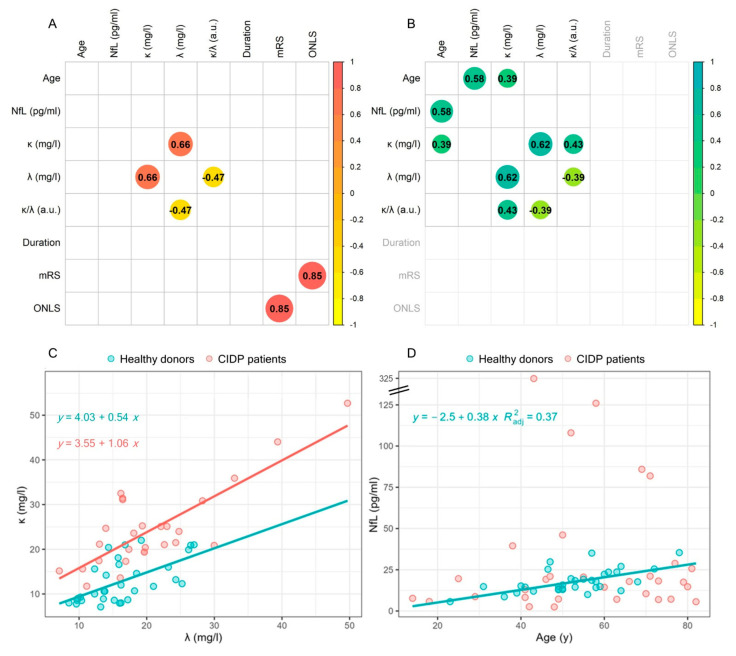
Heat map of Spearman’s correlation coefficients between NfL, FLCs (k and λ), and patient parameters for CIDP (**A**) and healthy subjects (**B**). All the reported correlations are statistically significant (*p* < 0.05). Linear regression analysis of kFLCs versus λFLCs (**C**). Linear regression analysis of NfL versus age of patients (**D**).

To identify potential associations among circulating NfL levels, FLC levels, and clinical scores, we conducted a correlation analysis using Spearman’s correlation coefficient (Figure 3). The correlogram revealed no significant correlations between NfL levels and any of the investigated variables in CIDP patients. However, a robust positive correlation was observed between k_FLC_ and λ_FLC_ (ρ = 0.66, *p* = 3.7 × 10^−5^). Furthermore, associations were identified between λ_FLC_ levels and the k_FLC_/λ_FLC_ ratio (ρ = −0.47, *p* = 0.006) and between the mRS and ONLS (ρ = 0.85, *p* = 7.7 × 10^−10^), two scores employed to assess the disability rate of patients (these latter correlations can be considered trivial and/or expected because of the mathematical definitions and, therefore, are not further discussed). Notably, no significant correlations were detected between circulating levels of NfL and FLCs and any of the measured clinical scores evaluating disease severity.

Consistent with the results from patients, a positive correlation was observed between k_FLC_ and λ_FLC_ in controls (Figure 2B, ρ = 0.62, *p* = 1.0 × 10^−4^). Of note, in contrast to CIDP patients, we observed positive correlations between the healthy controls’ age and k_FLC_ (ρ = 0.39, *p* = 0.025) and between age and NfL (ρ = 0.58, *p* = 6.0 × 10^−4^).

To gain a deeper understanding of k_FLC_ and λ_FLC_ behavior, we conducted a multivariate linear regression analysis of k_FLC_ levels, considering λ_FLC_ and group membership (CIDP patients or controls, coded as 0 and 1). The model incorporates interactions and can be described by the following equation: *k_FLC_* = *a* · *λ_FCL_* + *b* · *CIDP* + *cλ_FCL_* · *CIDP* + *q*, where q represents the intercept, and a, b, and c are the coefficients for λ_FLC_, CIDP, and the interaction term, respectively. The results are summarized in Table 2, highlighting high significance (*p* < 2.2 × 10^−16^) and substantial adjusted R-squared value (Adj. R^2^ = 0.80). Notably, as discussed later, the significance of λ_FLC_ coefficient (*p* = 0.006) and interaction term (*p* = 0.022) were observed. The regression model is visually depicted in Figure 3C, showing a good fit of data points. The best regression equations for the two groups, derived from Table 2, are superimposed on each plot.

In Figure 3D, a scatter plot analysis of sNfL levels as a function of age is presented for controls (green dots) and CIDP patients (red dots). A regression analysis was performed on controls alone, as including patients resulted in a clear deviation from the linear model assumption. The findings confirm the presence of a distinct increase in sNfL levels with age in control subjects. These results are summarized in Table 3, and the best-fitting regression line (continuous line) along with the corresponding equation is superimposed on the plot.

Conversely, as expected from Figure 3B, sNfL levels in patients display a scattered pattern as a function of age, with no clear trend, and are strongly influenced by the presence of particularly elevated sNfL values in selected CIDP patients.

## 3. Discussion

In this paper, we present a comparative study of serum neurofilament light chain and free light chain levels in CIDP patients and controls. We observed a significant increase in k_FLC_ and λ_FLC_ levels, as well as in the k_FLC_/λ_FLC_ ratio in CIDP patients compared to controls. However, no significant differences were noted in neurofilament levels, likely due to the patients undergoing pharmacological treatments to limit neuronal damage. Considering that the majority of patients enrolled in the present study were treated with intravenous immunoglobulin, this aligns with the results of Kapoor et al. [6], demonstrating the effectiveness of this treatment type in normalizing NfL values.

Conversely, the data in Figure 1 allow us to hypothesize that the underlying inflammatory processes persists, contributing to the observed alterations in FLC levels. This suggests that, in treated patients, NfL levels are well suited for monitoring treatment efficacy, but they may not be as effective for assessing the overall inflammatory state of patients, which could be better described by circulating FLC levels. These molecular chains are indeed strongly influenced by various immune system responses, associated with different autoimmune and chronic disease conditions [13]. However, the specific role of FLCs in the development of brain inflammation is not yet fully understood and is currently under debate in the literature [17].

In this context, there is a growing research effort to assess the effectiveness of these filaments as diagnostic, prognostic, and disease-monitoring markers in neuropathologies. For instance, Kaya et al. recently found a significant difference in k_FLC_/λ_FLC_ ratios (detected via MALDI-TOF-MS and chromatography) between Alzheimer’s disease (AD) patients and controls (mean difference −0.409; *p* < 0.001), suggesting potential clinical utility for the ratio as an AD diagnostic tool [18]. It is interesting to note that the mean difference reported by the authors is of the same order as the median difference measured in the present study (median difference 0.34, *p* < 0.001) but it goes in the opposite direction, an interesting finding that deserves further detailed investigation. Furthermore, Konen and collaborators investigated k_FLC_ levels in the serum and CSF of patients with newly diagnosed MS or CIS, both pre- and post various immune therapies. The authors showed that methylprednisolone treatment notably reduces serum k_FLC_ levels, while other therapy types have minimal effect [19]. These findings align with our study, which showed elevated FLC values in CIDP patients, only two of whom received purely steroid treatments.

In a comparable manner, numerous studies have indicated the strong diagnostic accuracy of the k_FLC_ isotype in the CSF for discriminating patients with multiple sclerosis from those with other neurological diseases, which also presents significant methodological advantages compared to the identification of oligoclonal bands (OCBs) [5,7,11].

Regarding the pivotal role of k_FLC_ as a biomarker of neurodegeneration, the multivariate analysis reported in Figure 3C enables us to formulate mechanistic hypotheses, seeking to elucidate the relationship between this parameter and λ_FLC_ levels in patients. While a direct proportionality between k_FLC_ and λ_FLC_ was indeed observed in both groups, the presence of a significant interaction term (Table 2) suggests a different slope in the two populations. Specifically, in CIPD patients, k_FLC_ levels increase at a faster rate than in controls as a function of λ_FLC_. One possible explanation for these findings can be derived from considering the different structures of kappa and lambda, with the former being a monomer, while the latter tends to dimerize. Concerning MS, Arneth et al. hypothesized that λ_FLC_ dimers may have a reduced propensity to cross the blood–CSF barrier compared to monomeric k_FLC_ [10]. If this same mechanism was confirmed in our case, it could potentially explain the observed behavior in Table 2 and Figure 3: the more severe the inflammation in the peripheral nervous system (PNS), the higher the levels of both of these markers in the CSF. However, due to their smaller size, k_FLC_ might exhibit greater permeability to the blood. Consequently, this would result in an additional contribution to k_FLC_ serum levels, which would increase proportionally with the severity of the disease.

Finally, in Figure 3D, we demonstrate an increase in NfL levels in the serum of control subjects with aging. Our findings are consistent with those of Khalil et al. regarding the association of sNfL with age and subclinical brain changes. Their study, conducted on a population-based cohort, revealed an increase and greater variability in sNfL levels in individuals over 60 years, suggesting accelerated neuronal injury possibly linked to underlying comorbid conditions. The strong correlation of sNfL with brain volume changes emphasizes its potential as a marker for assessing subclinical brain damage [20].

However, in our study, the same trend was not evident in the diseased subjects, where the combined effect of the presence of the condition and the treatment appears to be the cause of significant fluctuations in neurofilament levels, surpassing the age-related effect by a large margin, and making it no more observable. These results contribute to strengthening the previously discussed hypothesis formulated by Khalil and co-workers on the role of comorbidities in enhancing the variability of NfL levels.

## 4. Materials and Methods

### 4.1. Patient Recruitment and Clinical Assessment

This study was conducted in accordance with the principles outlined in the Declaration of Helsinki, and it received approval from the Fondazione Policlinico Gemelli IRCCS Ethics Committee (Prot. 23255/16 ID 1229). Informed consent was obtained from all participating patients before their inclusion in the study, ensuring adherence to ethical guidelines.

Blood samples were prospectively collected from patients with a diagnosis of CIDP attending the Neurology Department of Fondazione Policlinico Universitario Agostino Gemelli IRCCS in Rome, Italy. The diagnosis of CIDP was based on the EAN/PNS criteria from 2021 [2]. All patients underwent treatment for the management of CIDP. Particularly, in patients undergoing monthly IVIg or SCIg therapy, blood samples were collected immediately prior to one of the drug administrations. Control samples were collected from 32 sex- and age-matched healthy volunteers without evidence of any neurologic or neuromuscular disease.

All patients underwent a comprehensive neurological evaluation by an expert neurologist and several measures for disability rate were assessed, including mRS and ONLS in its total, leg, and arm sections.

### 4.2. Laboratory Testing

The quantification of free light chain levels (κ and λ) from the serum of both patients and controls followed previously established methods [21,22]. Briefly, the collected samples were centrifuged at 2500× *g* for 10 min. The serum was then divided into aliquots and subsequently frozen at −80 °C for storage until analysis. Thawing of samples occurred once, with maintenance at room temperature, and immediate analysis thereafter. The analysis was conducted by an independent operator blinded to the clinical history of the samples, ensuring an unbiased assessment.

Each sample was analyzed using OPTILITE (The Binding Site, Birmingham, UK) analyzers, in strict accordance with the prescribed protocols of the manufacturer. The analytical sensitivity and specificity of Freelite assays, the benchmark method, was achieved by the use of latex particles coated with affinity-purified polyclonal antibodies. FLCs were assessed by means of turbidimetric assay (Freelite Human Kappa and Lambda Free Kits, The Binding Site, Birmingham, UK) and performed with the Optilite^®^ instrument (The Binding Site). The immunoassay consisted of two separate measurements for free κ (normal range = 3.3–19.4 mg/L) and free λ (normal range = 5.7–26.3 mg/L). A ratio of κ/λ < 0.26 or >1.65 is abnormal, according to the manufacturer’s recommendations. Calibrators and controls were provided by the manufacturer and consisted of stabilized human sera containing polyclonal λ- and κ-FLC; calibrators and controls were diluted to the appropriate concentrations for serum determinations, following the manufacturer’s instructions.

Serum NfL concentration was quantified utilizing the Simple Plex™ cartridge-based assay on the Ella™ platform (ProteinSimple, San Jose, CA, USA), following the detailed instructions provided by the manufacturer. Thawed samples underwent a single assay process under blinded conditions, all within a solitary batch. Serum dilutions were executed when necessary, adhering strictly to the recommended procedures of the manufacturer.

### 4.3. Statistical Analysis

Statistical analyses were performed using the R software package (version 4.0.2, R Foundation, Vienna) [23]. Categorical data were presented in terms of absolute frequencies and/or percentages. Continuous variables were assessed for normality through visual inspection of the qq-plot and trough the Shapiro–Wilk test. Tabular data were created with the gtsummary software package (version 1.7.2) [24]. As significant deviations from normality were observed in selected parameters, all the investigated continuous variables were reported as the median and the corresponding Interquartile Range (IQR), defined as Q3-Q2. Consequently, comparisons between two independent groups were performed using the Wilcoxon Mann–Whitney test for two independent samples.

The Spearman’s coefficient was utilized to assess the presence of correlations among sNfL levels, sFLC levels, demographic data (age), and clinical data (disease duration, as well as clinical scales including mRS: ONLS—arm score, ONLS—leg score, ONLS—total score). Correlation data were visualized using correlation maps [25], in accordance with previous studies [26]. In brief, the blank positions in the correlation maps correspond to non-statistically significant correlations according to power analyses (the significance level was set at *p* < 0.05). Significant correlations are represented using solid dots. A dual visual scale was employed to indicate the strength of the correlation; the larger and simultaneously the more intense the color, the stronger the correlation value. The direction of the correlation, positive or negative, was denoted by a color scale. Two distinct scales were used for controls and pathological subjects: for the former, correlations followed a scale from yellow (negative correlation) to green (positive correlation), and for the latter, from yellow (negative correlation) to red (positive correlation). In this analysis, quantitative clinical score values were included for pathological subjects, which, however, were not available for healthy subjects. Univariate and multivariate linear regression analyses were conducted using the R software package to better understand the potential contribution of age and the relationships among selected biomarkers. Data were visualized with the R software package ggplot2 and corrplot [27].

## 5. Conclusions

Our findings may support the utility of serum NfL levels in evaluating neuronal damage in CIDP patients. However, implementing the study by collecting more samples from CIDP patients both before and after therapy is necessary to define the real usefulness of serum NfL in monitoring this condition. Additionally, the study underscores the potential of kappa and lambda FLCs, along with their ratio, as promising circulating markers of neuroinflammation, a topic currently subject to intense debate. Overall, these results could significantly contribute to the development of personalized therapeutic approaches, ultimately leading to improved treatment outcomes for patients.

## Figures and Tables

**Table 1:** Clinical, demographic and laboratory characteristics of the enrolled subjects.

Variable	N	CIDP, N = 32 ^1^	CTRL, N = 32 ^1^	*p*-Value ^2^
**Age**	64	56 (43, 72)	53 (48, 59)	0.4
**Sex**	64			>0.9
F		13/32 (41%)	13/32 (41%)	
M		19/32 (59%)	19/32 (59%)	
**NfL (pg/mL)**	64	18 (8, 26)	15 (13, 20)	0.9
**κ (mg/L)**	64	23 (19, 31)	11 (9, 16)	<0.001
**λ (mg/L)**	64	19 (16, 24)	16 (13, 19)	0.022
**κ/λ (a.u.)**	64	1.12 (1.01, 1.51)	0.78 (0.58, 0.92)	<0.001
**mRS**	32			
<4		28/32 (88%)		
≥4		4/32 (12%)		
**ONLS—arm score**	32			
<3		19/32 (59%)		
≥3		13/32 (41%)		
**ONLS—leg score**	32			
<4		29/32 (91%)		
≥4		3/32 (9.4%)		
**ONLS**	32			
<7		28/32 (88%)		
≥7		4/32 (12%)		

^1^ Median (IQR); n/N (%). ^2^ Wilcoxon rank sum test; Pearson’s Chi-squared test; Fisher’s exact test.

**Table 2:** Multivariate linear regression analysis of k_FLC_ levels as a function of λ_FLC_, group membership (CIDP or controls, codified as 0 and 1), and interaction term.

Variable	Coefficient	SE	*p*-Value
Intercept	4.03	3.24	0.22
λ	0.54	0.19	0.006
CIDP	−0.48	3.92	0.9
λ*CIDP	0.52	0.21	0.02
F(3,59) = 81.7; *p*-value < 2.2 × 10^− 16^; Adj. R^2^ = 0.80			

**Table 3:** Univariate linear regression of NfL levels as a function of age in healthy control subjects.

Variable	Coefficient	SE	*p*-Value
Intercept	−2.46	4.67	0.60
Age	0.38	0.09	0.0001
F(1,30) = 19.4; *p*-value < 1.2 × 10^−4^; Adj. R^2^ = 0.37			

## Data Availability

The data that support the findings of this study are available from the corresponding author, upon reasonable request.
